# Supplementary material for: Targeted gene editing and near-universal cDNA insertion of CYBA and CYBB as a treatment for chronic granulomatous disease
Source: Nat Commun. 2025 Aug 12;16:7475. doi: 10.1038/s41467-025-62738-2 (PMC12343970; doi:10.1038/s41467-025-62738-2)
Supplement: Supplementary file 1 — Supplementary Information [file 41467_2025_62738_MOESM1_ESM.pdf]

Targeted gene editing and near-universal cDNA insertion of *CYBA* and *CYBB* as a treatment for chronic granulomatous disease

Supplementary Information

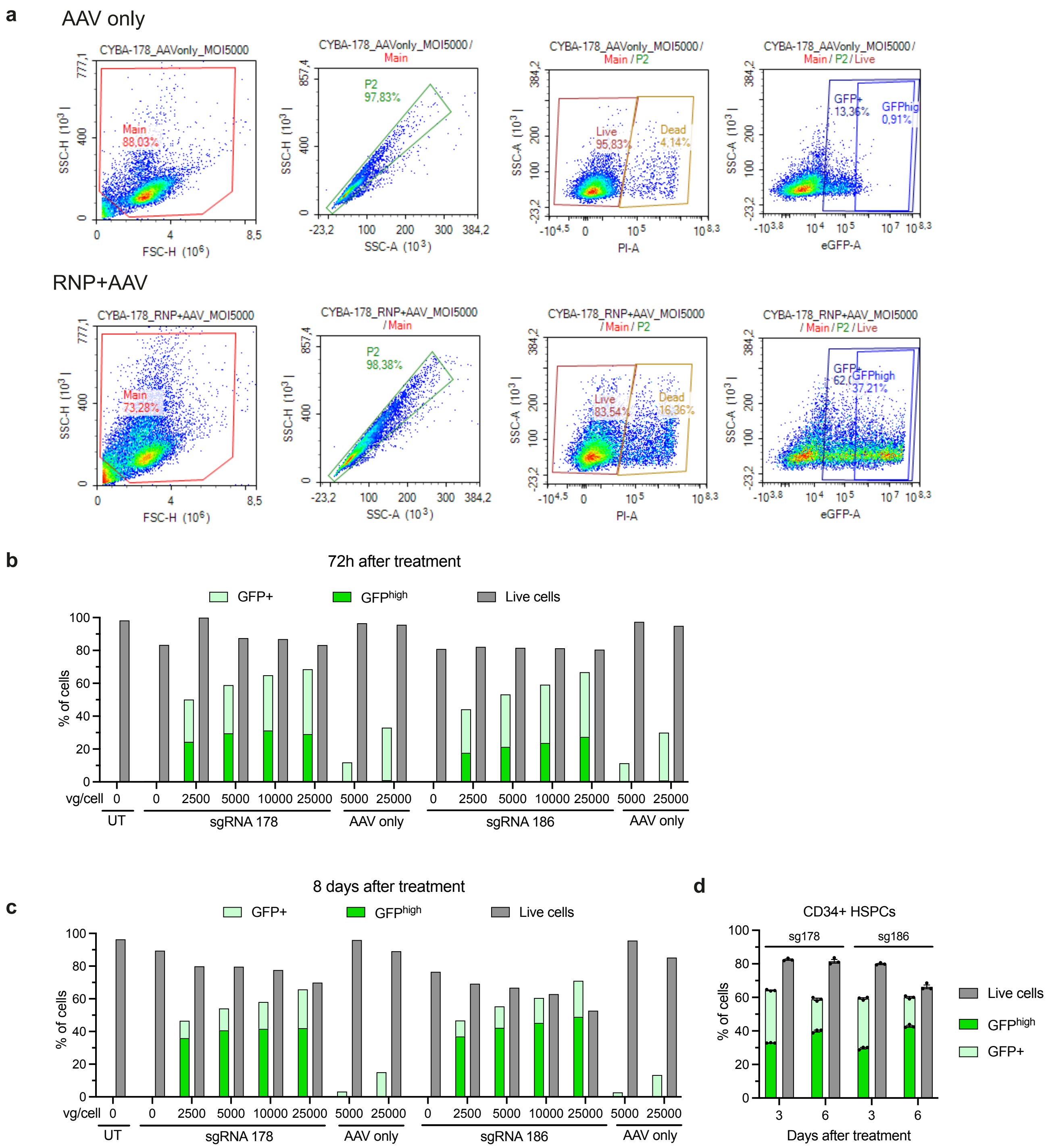

Supplementary Figure 1. Functional titration of SFFV-GFP rAAV6 in CD34+ HSPCs. a Gating strategy for quantification of targeted insertion of SFFV-GFP cassette in CD34+ HSPCs. Targeted insertion was determined as the percentage of GFP^{high} cells. **b** Functional titration of rAAV6 in CD34+ HSPCs using sg178 and sg186 measured 72h after nucleofection. **c** Functional titration of rAAV6 in CD34+ HSPCs using sg178 and sg186 measured 8 days nucleofection. **d** Targeted insertion rates in CD34+ HSPCs using 5000 vector genomes/cell (vg/cell) measured 3 and 6 days after nucleofection. Source data are provided as a Source Data file.

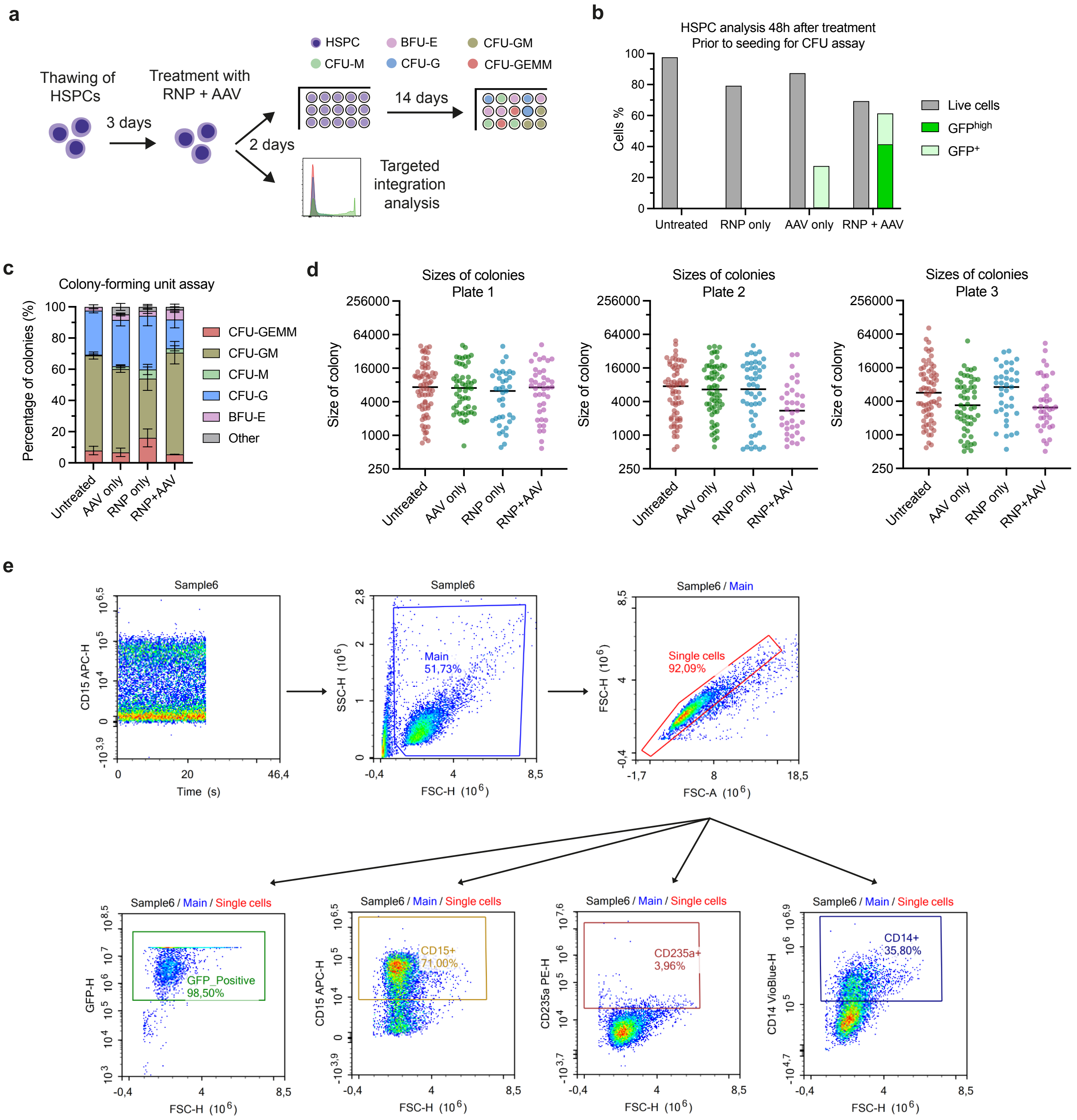

Supplementary Figure 2. Flow cytometry-based CFU assay. a Schematic overview of the flow cytometry-based colony-forming units (CFU) assay. **b** Targeted insertion rates in CD34+ HSPCs prior to seeding for the CFU assay measured 2 days after treatment. **c** Result of CFU assay showing CFU types as percentage of total. **d** The sizes of colonies across all three replicate plates were measured as the total number of cells in each colony. **e** Gating strategy used for analysis of the CFU assays. Source data are provided as a Source Data file.

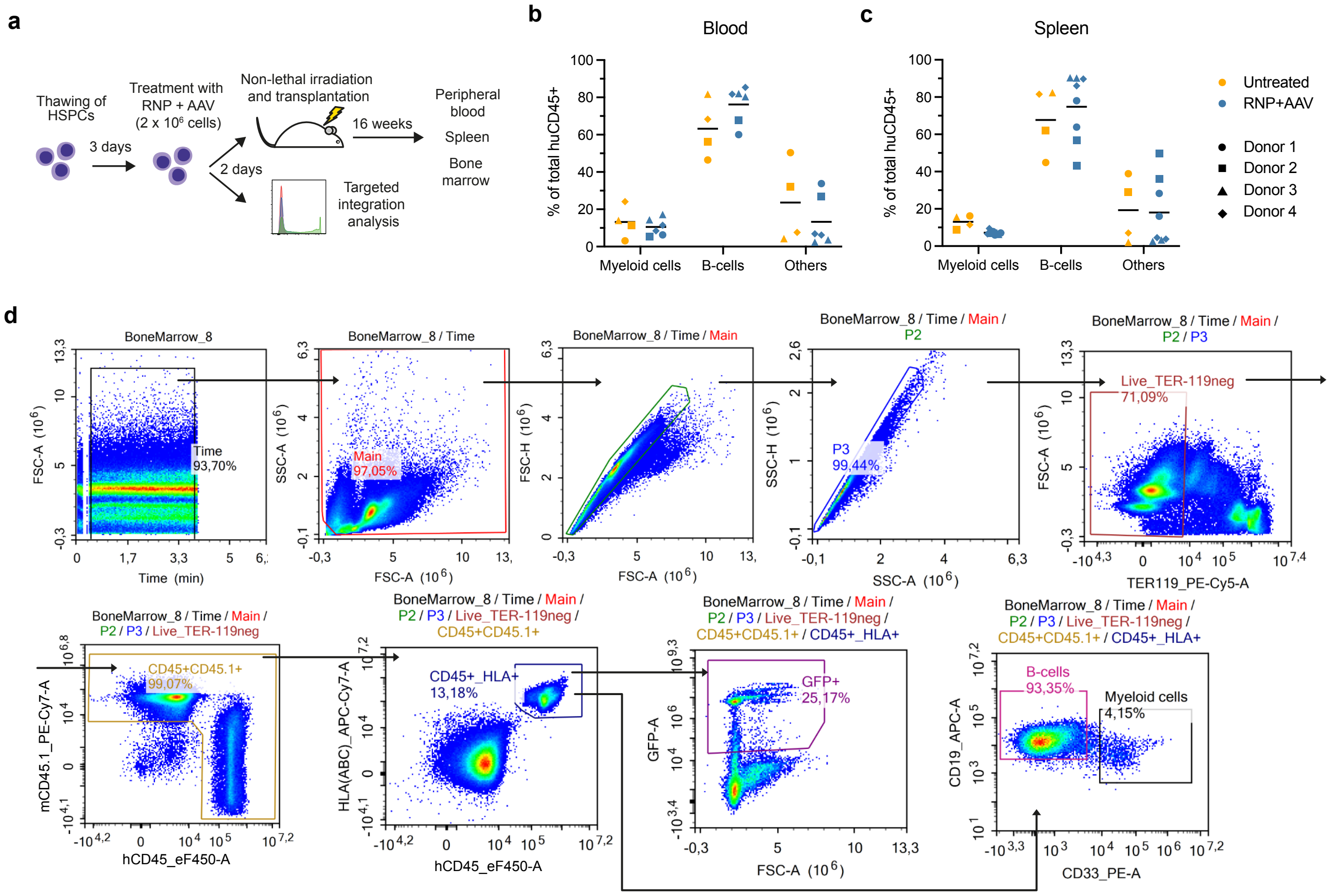

Supplementary Figure 3. Engraftment of gene edited HSPCs in immunodeficient NOG-F mice. a Experimental setup of xenotransplantation studies. We transplanted the total outgrowth of 2x10⁶ nucleofected cells. **b-c** Distribution of lineages in the human graft in the blood (b) and spleen (c). Experiments were conducted using HSPCs from 4 individual healthy donors. **d** Representative gating strategy for the terminal analysis of engrafted immunodeficient mice. Source data are provided as a Source Data file.

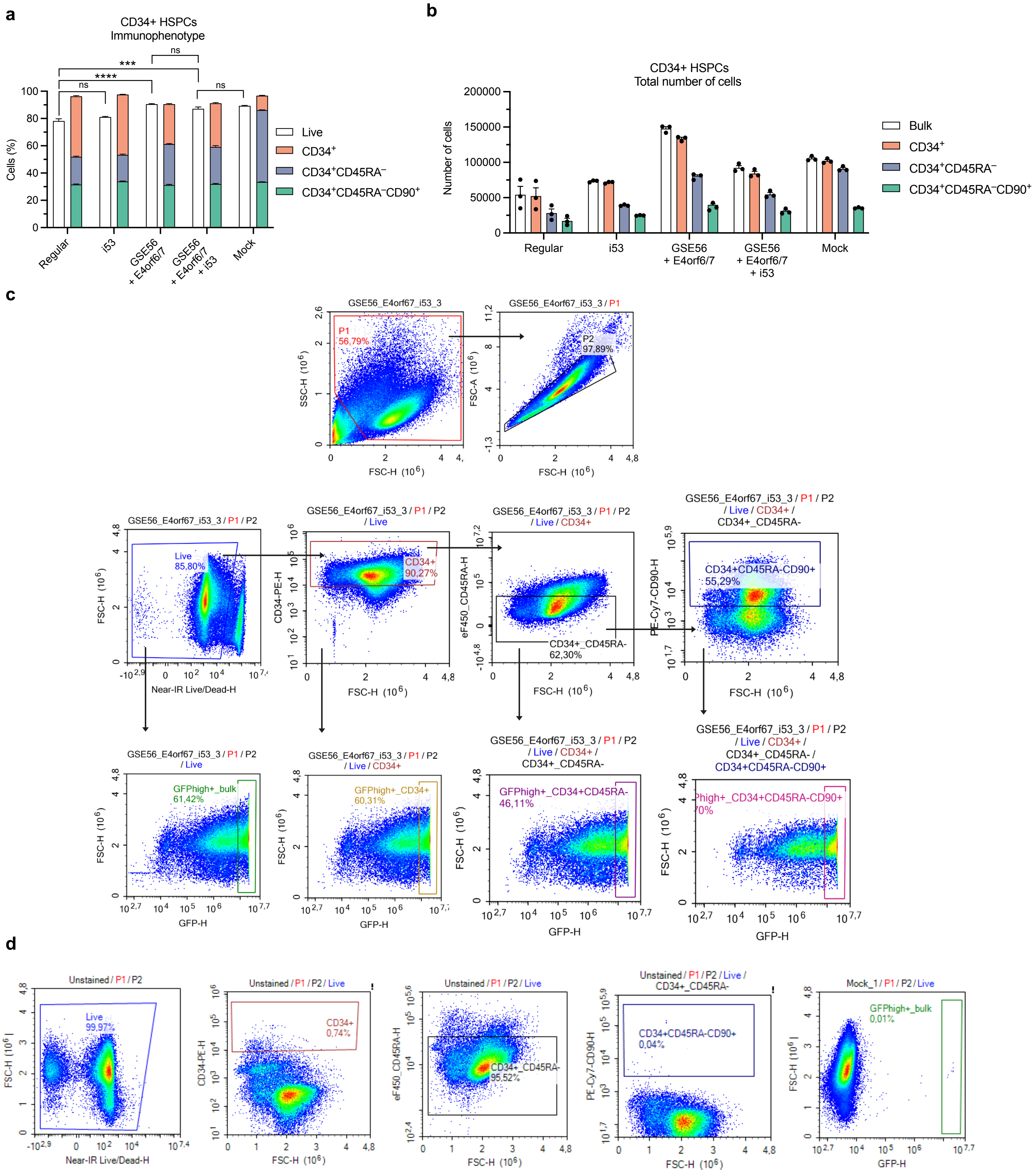

Supplementary Figure 4. Optimizing gene editing in HSPCs using mRNA-transcripts a Distribution of different HSPC subpopulations using i53, GSE56 and Ad5-E4orf6/7 mRNA transcripts measured by flow cytometry 4 days after nucleofection. **b** Total cell numbers in different HSPC subpopulations using i53, GSE56 and Ad5-E4orf6/7 mRNA transcripts measured by flow cytometry 4 days after nucleofection. **c** Gating strategy for analysis of targeted integration levels in different HSPC subpopulations. **d** Unstained or biological controls that were used to place gates. Data represented as mean (n = 3) +/- standard deviation. Statistical significance was determined by one-way ANOVA with Tukey's multiple comparisons test. For the CFU assay, total colony numbers were used for ANOVA test. (*); $p<0.05$, (**); $p<0.01$, (***); $p<0.001$, (****); $p<0.0001$. Source data are provided as a Source Data file.

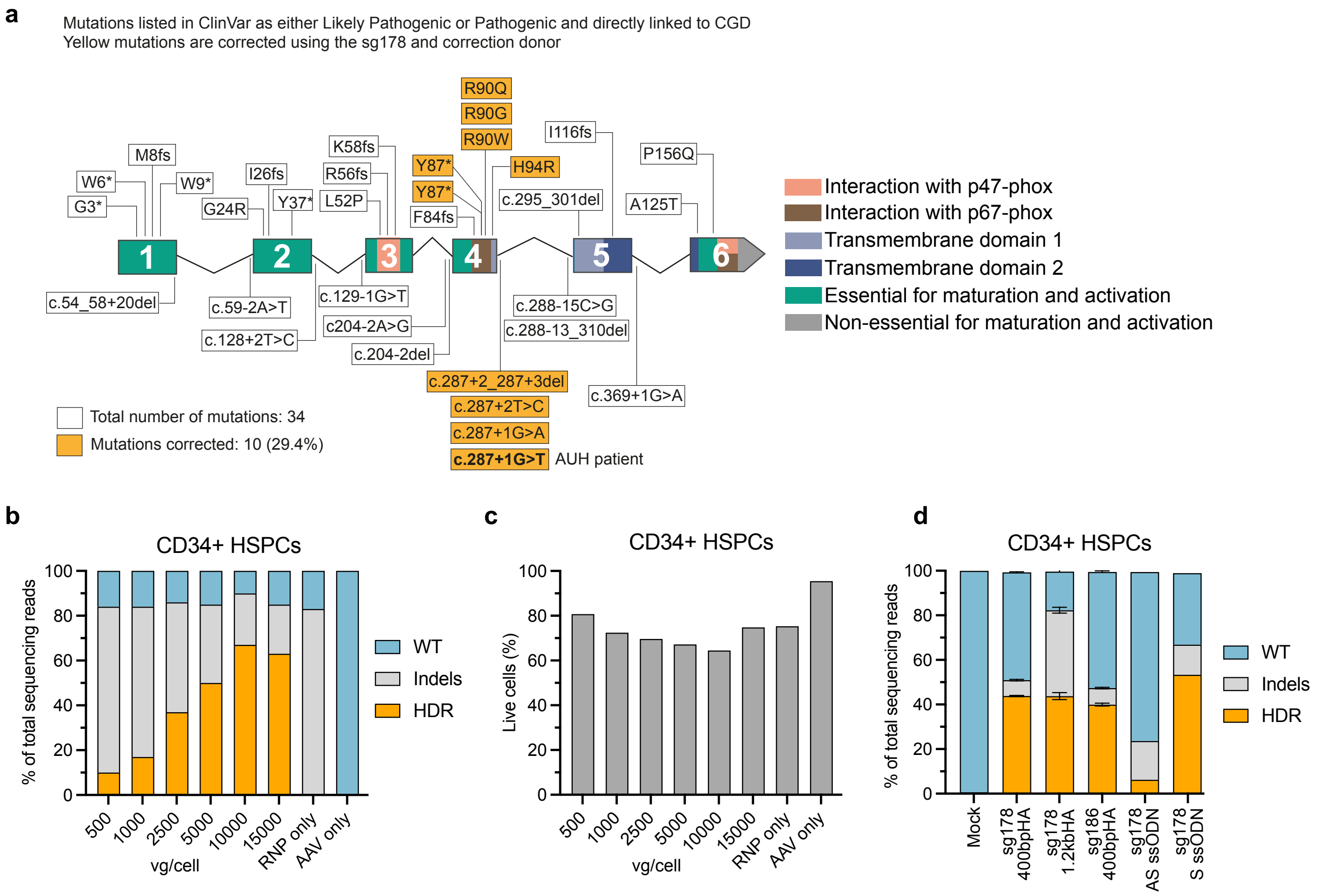

Supplementary Figure 5. Gene editing of *CYBA* c.287+1G>T variant. a ClinVar variants that are potentially correctable using the *CYBA* HDR repair template. **b** Functional titration of the correction donor in CD34+ HSPCs from a healthy donor. **c** Viability data of cells from (b). **d** Gene editing levels when using a HDR repair template with 1.2 kb homology arms as well as gene editing levels using both sense (S) and antisense (AS) ssODNs. Source data are provided as a Source Data file.

a

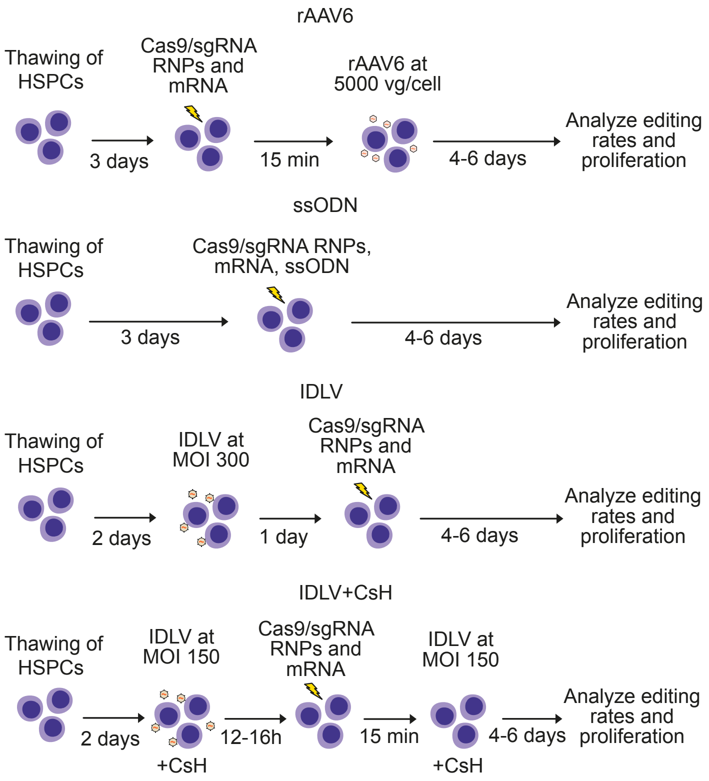

Supplementary Figure 6. Comparison of rAAV6, ssODN and IDLV as vehicle for donor delivery. a Overview of protocols used for HDR using rAAV6, ssODN and IDLV for donor delivery.

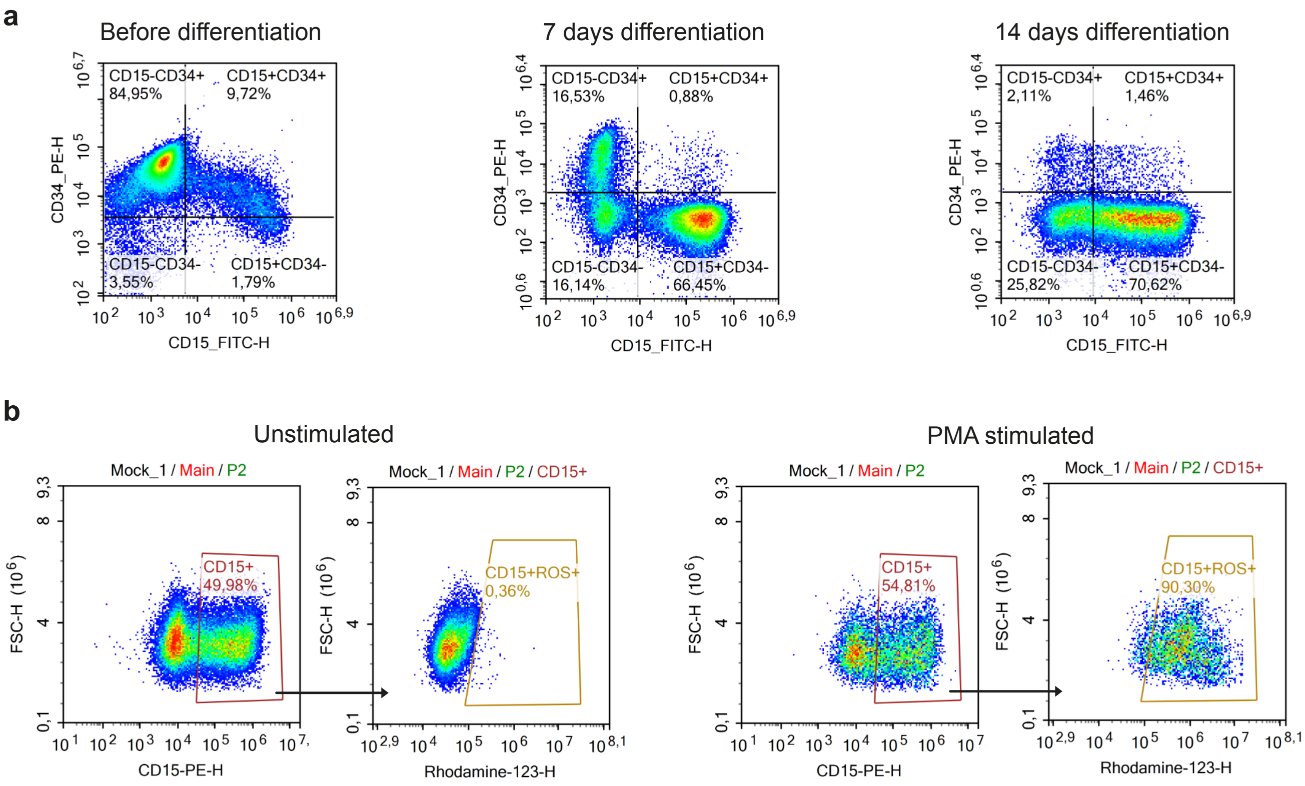

Supplementary Figure 7. Oxidative burst assay in differentiated granulocytes. a Representative flow cytometry plots used to validate the differentiation of granulocytes from CD34+ HSPCs. **b** Gating strategy used for the oxidative burst assay. Gates for ROS+ cells were set based on unstimulated cells.

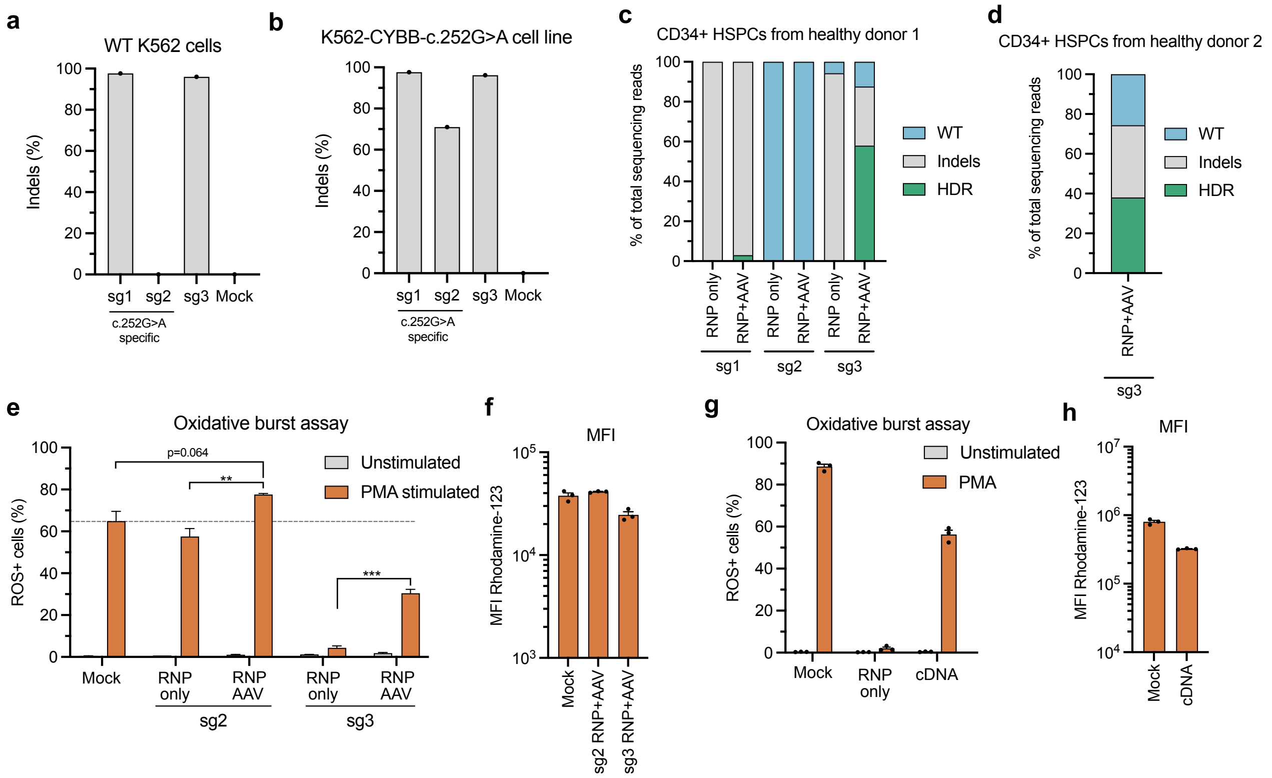

Supplementary Figure 8. Gene editing of *CYBB* c.252G>A variant. a Indel formation of *CYBB* sgRNAs in wild-type K562 cells. sg1 and sg2 were designed to be allele-specific. **b** Indel formation of *CYBB* sgRNAs in a K562 cell line homozygous for the *CYBB* c.252G>A variant. **c** RNP/AAV-mediated gene editing in CD34+ HSPCs from a healthy donor. **d** RNP/AAV-mediated gene editing in CD34+ HSPCs from a separate healthy donor. **e** Non-normalized plot of Fig. 3e showing absolute ROS+ cells. **f** Median fluorescent intensity (MFI) of Rhodamine-123 in ROS+ cells from (e). **g** Non-normalized plot of Fig. 3j showing absolute ROS+ cells. **h** MFI of Rhodamine-123 in ROS+ cells from (g). Source data are provided as a Source Data file.

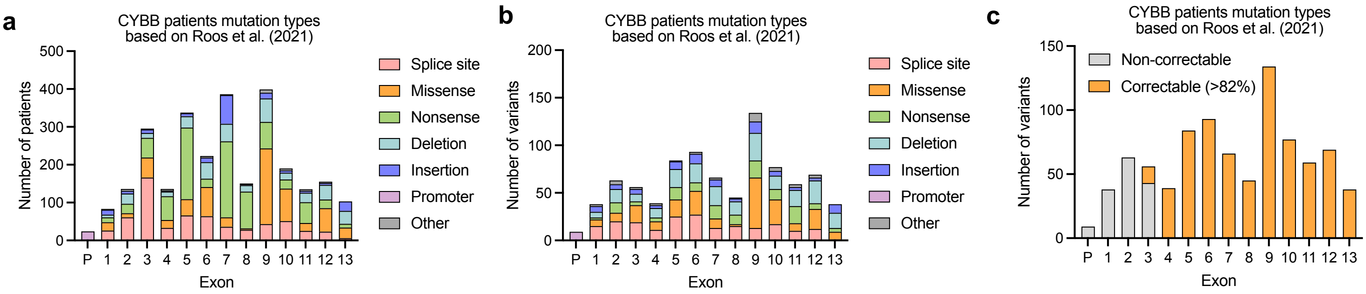

Supplementary Figure 9. X-CGD patients potentially treatable by the cDNA strategy. a Number of patients listed in Roos et al. (2021)[1] with each type of mutation. **b** Number of unique genetic variants listed in Roos et al. (2021). **c** Number of unique genetic variants from Roos et al. (2021) that could potentially be corrected by the Ex3-13 cDNA insertion strategy. The calculation of correctable variants does not take into account X-CGD cases caused by large deletions of part of chromosome X, such as does found in patient with McLeod syndrome. Source data are provided as a Source Data file.

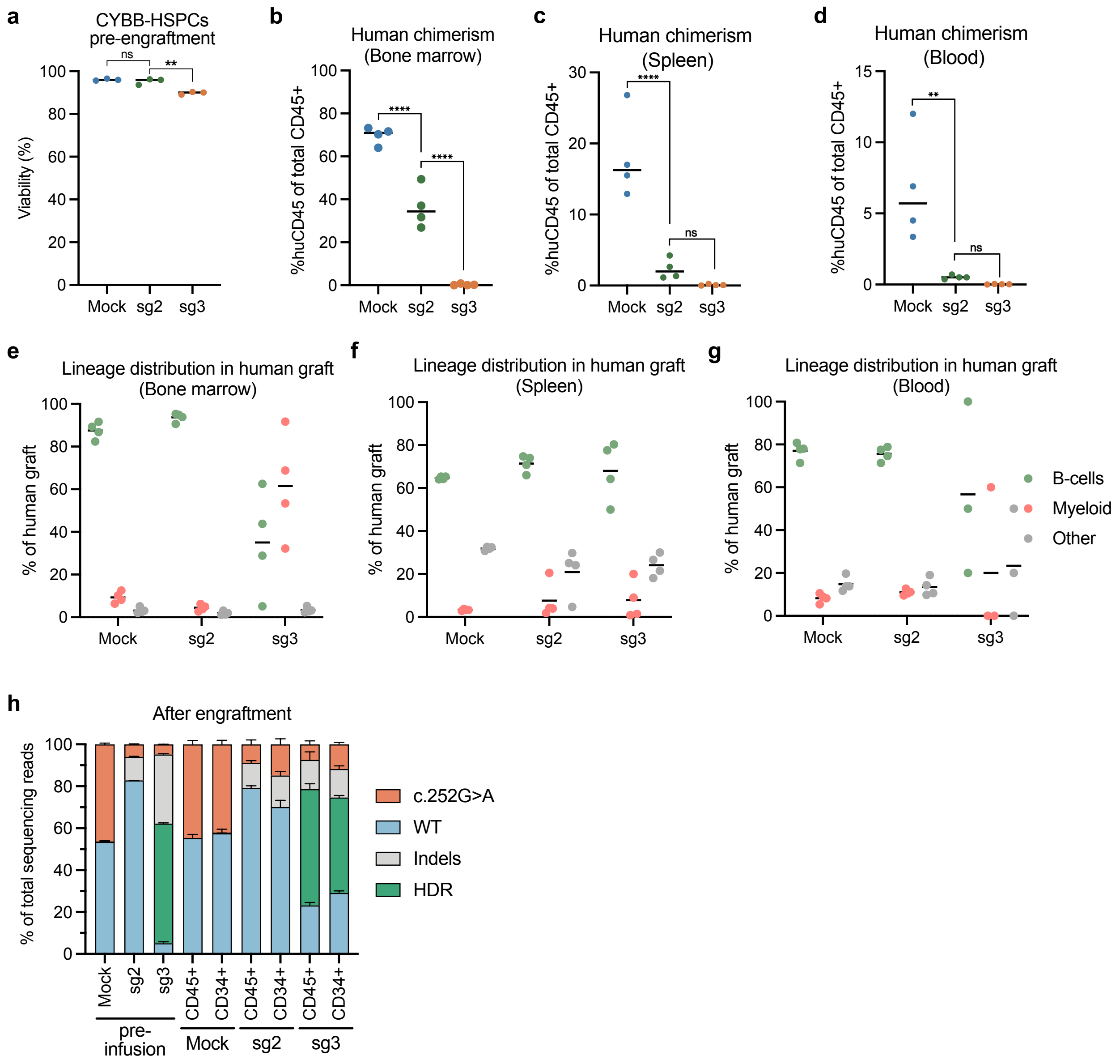

Supplementary Figure 10. Engraftment of CYBB-HSPCs. a Viability of gene edited CYBB-HSPCs prior to engraftment measured 48h after nucleofection. **b-d** Human chimerism in the bone marrow (b), spleen (c) and peripheral blood (d) of NOG mice 16 weeks after injection. **e-g** Distribution of human $CD19^{+}$ B-cells and $CD33^{+}$ myeloid cells in the bone marrow (e), spleen (f) and peripheral blood (g) of NOG mice 16 weeks after injection. **h** Sequencing of $CD45^{+}$ and $CD34^{+}CD10^{-}$ cells sorted from the bone marrow of injected mice. Statistical significance was determined by one-way ANOVA with Tukey's multiple comparisons test. For the CFU assay, total colony numbers were used for ANOVA test. (*); p<0.05, (**); p<0.01, (***); p<0.001, (****); p<0.0001. Source data are provided as a Source Data file.

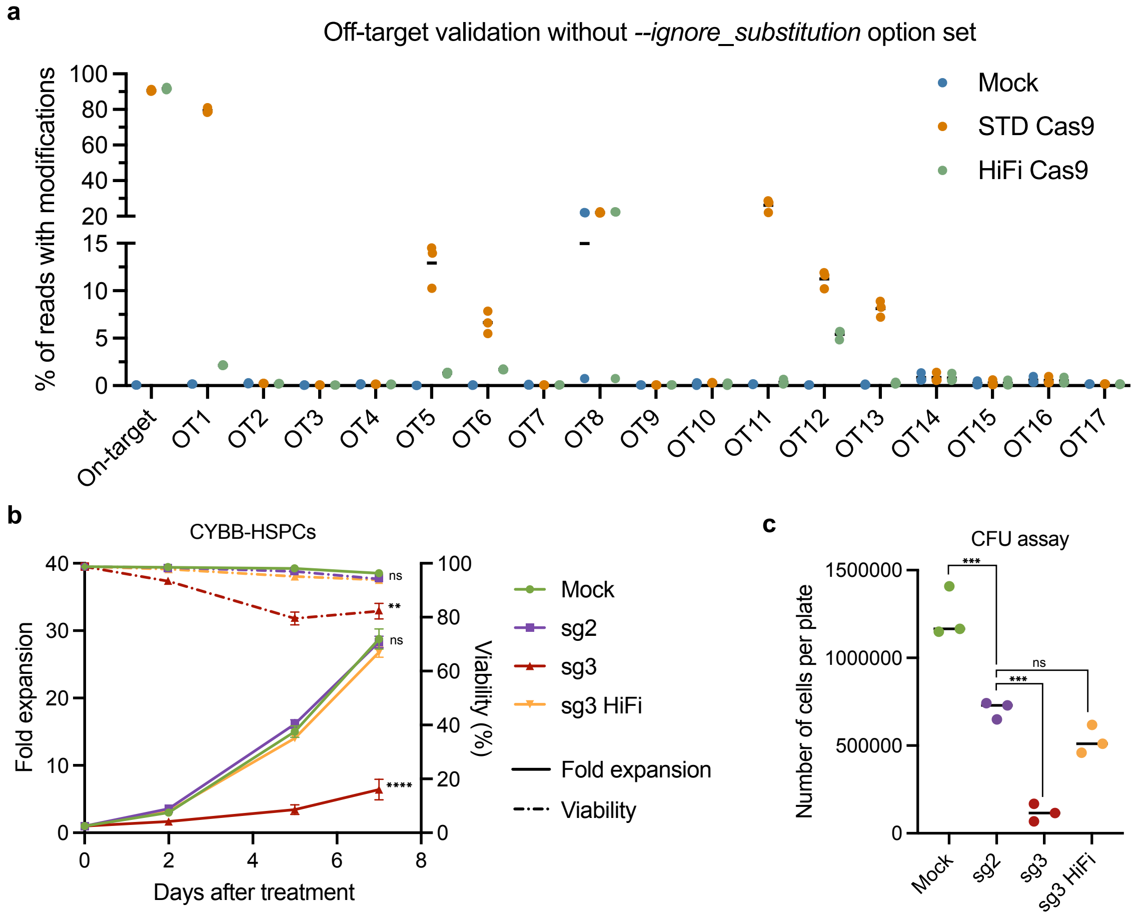

Supplementary Figure 11. **Off-target editing and cytotoxicity using HiFi Cas9. a** Validation of off-targets as in fig. 4b, but without the --ignore_substitution option set in CRISPResso2. **b** Proliferation data of CYBB-HSPCs comparing the use of HiFi Cas9 to standard (STD) Cas9 for correction of the *CYBB* c.252G>A variant. **c** The total number of cells in each CFU plate was measured at the end of the CFU assay. Statistical significance was determined by one-way ANOVA with Tukey's multiple comparisons test. For the CFU assay, total colony numbers were used for ANOVA test. (*); p<0.05, (**); p<0.01, (***); p<0.001, (****); p<0.0001. Source data are provided as a Source Data file.

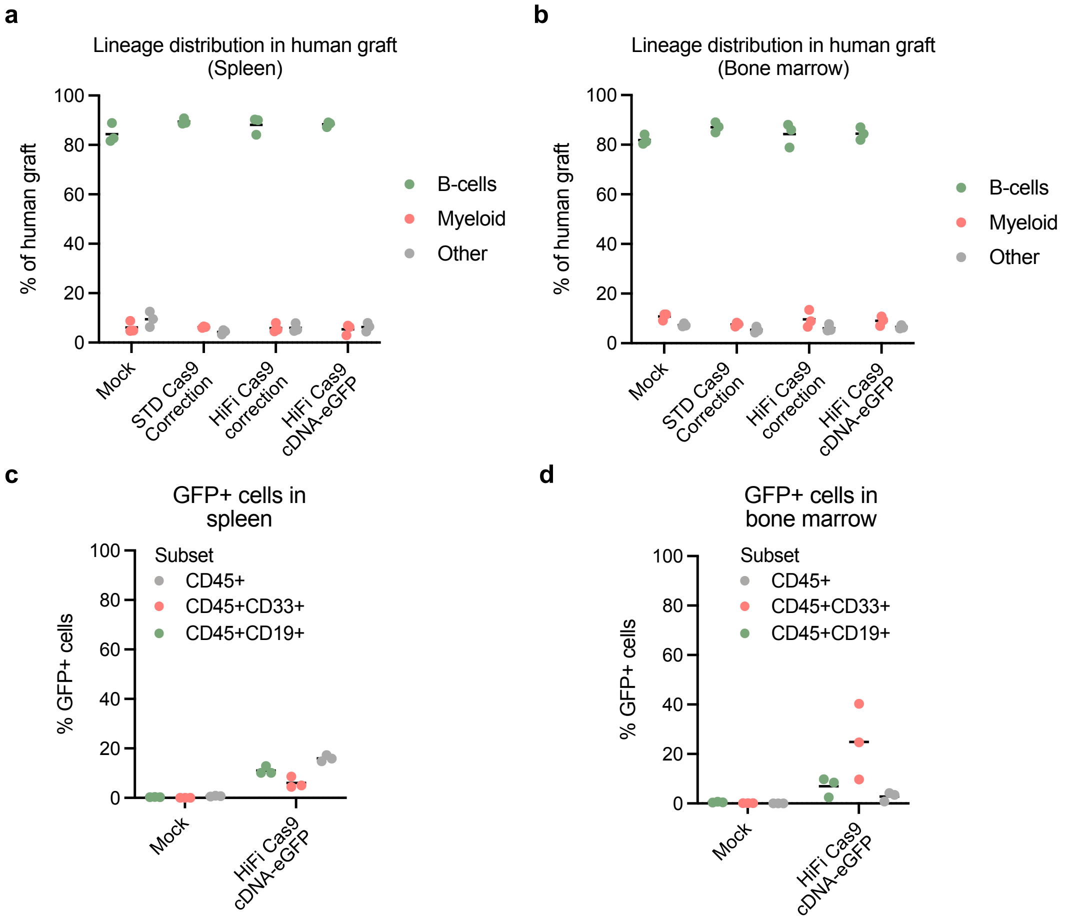

Supplementary Figure 12. Engraftment of CYBB-HSPCs edited using HiFi-Cas9. a-b Distribution of human CD19^{+} B-cells and CD33^{+} myeloid cells in the spleen (a) and bone marrow (b) of NOG mice 16 weeks after injection. **c-d** The percentage of GFP+ cells in spleen (c) and bone marrow (d) of NOG mice 16 weeks after injection. Source data are provided as a Source Data file.

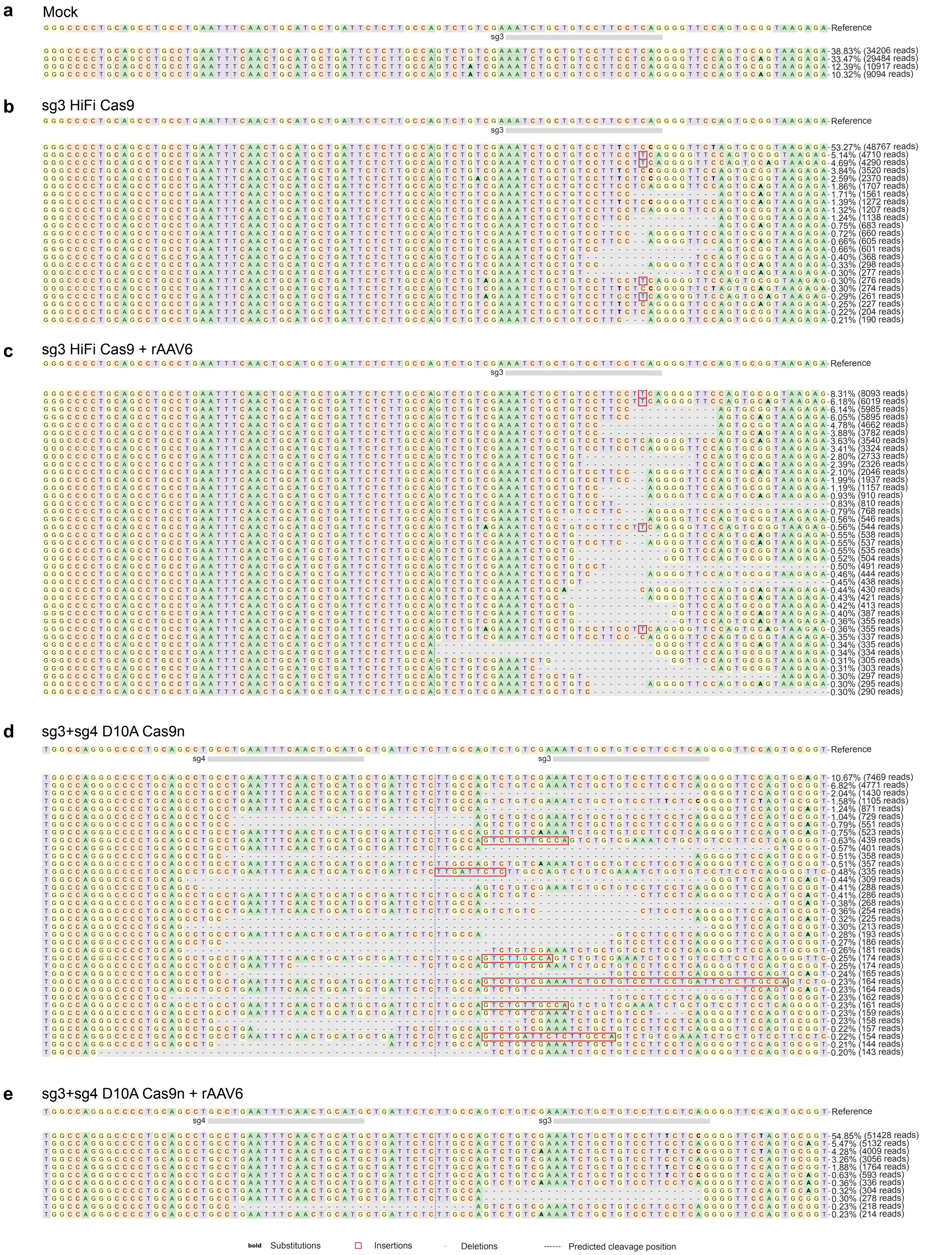

Supplementary Figure 13. Representative CRISPResso2 allele plots. a Mock. **b** sg3 HiFi Cas9 RNP only. **c** sg3 HiFi Cas9 + rAAV6 correction repair template. **d** sg3+sg4 D10A Cas9n RNP only. **b** sg3+sg4 D10A Cas9n + rAAV6 correction repair template.

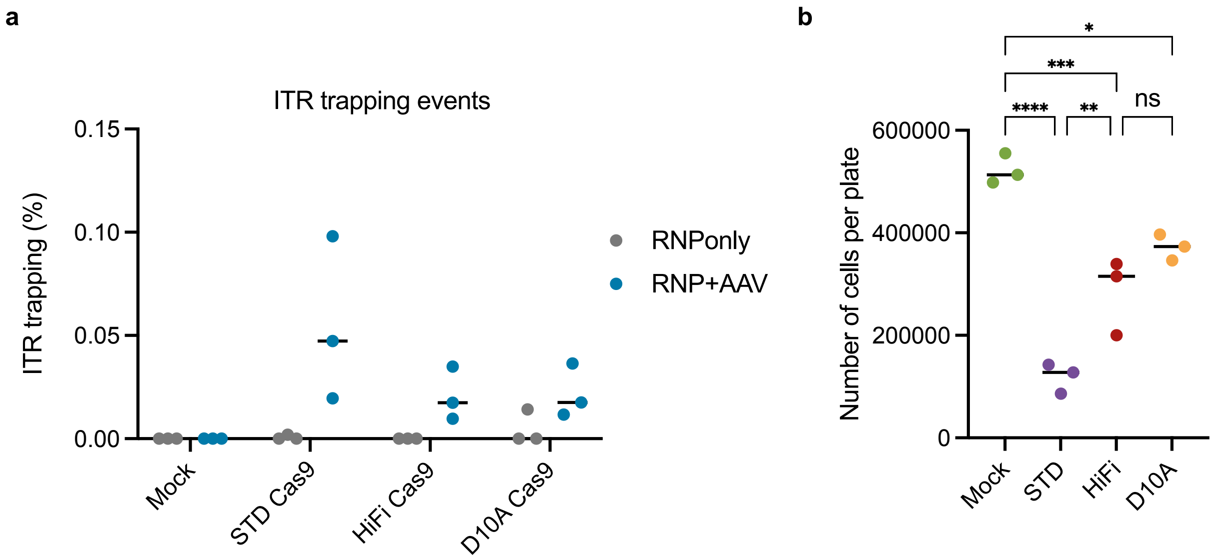

Supplementary Figure 14. D10A Cas9n-mediated gene editing of CYBB-HSPCs. a ITR-trapping events were determined as the percentage reads containing ≥20 bp insertions mapping to the ITRs of the AAV genome. **b** The total number of cells in each CFU plate was measured at the end of the CFU assay. Statistical significance was determined by one-way ANOVA with Tukey's multiple comparisons test. For the CFU assay, total colony numbers were used for ANOVA test. (*); p<0.05, (**); p<0.01, (***); p<0.001, (****); p<0.0001. Source data are provided as a Source Data file.

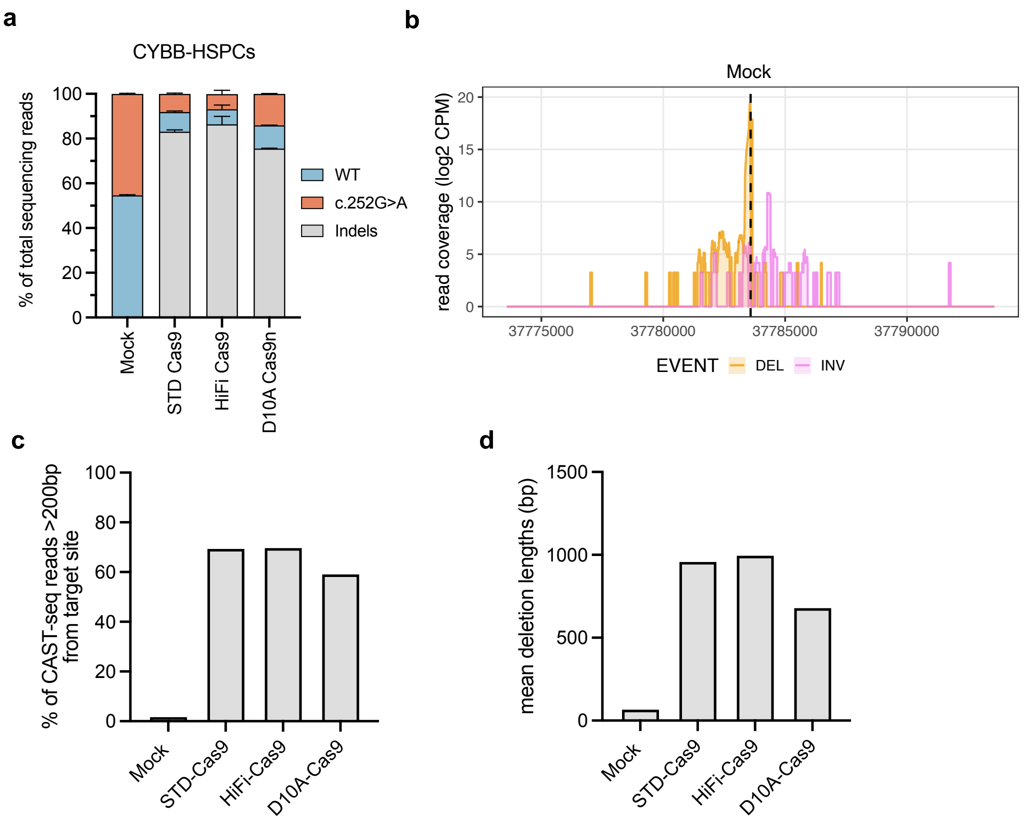

Supplementary Figure 15. On-target large deletions identified by CAST-seq. a On-target sequencing of CYBB-HSPCs used for CAST-seq analysis. **b** Coverage plot of untreated cells from CAST-seq showing all reads mapped to a 10 kb region of the on-target site. Deletions (DEL) are shown in orange and inversions (INV) are shown in purple. **c** The percentage of CAST-seq reads mapping >200 bp from the target site. **d** The mean length of deletions from (b). Source data are provided as a Source Data file.

Supplementary Table 1. Sequences of sgRNAs

Gene	sgRNA	sgRNA spacer sequence (5'-3')
CYBA	sg178	GGCCCGAACATAGTAATTCC
	sg186	TTACCAGGAATTACTATGTT
	sg187	TACCAGGAATTACTATGTTC
	sg221	CGTCCCGCACCCCCTCTAGG
CYBB	gRNA_c.252G>A	AACATTTTCTCTTACCGCAC
	sg1	TTACTGCACTGGAACCCCTG
	sg2	AACATTTTCTCTTACTGCAC
	sg3	AATCTGCTGTCCTTCCTCAG
	sg4	CATGCAGTTGAAATTCAGGC
	sg5	GACAGCAGATTTCGACAGAC

Supplementary Table 2. HDR templates

Donor	Sequence (5'-3')
SFFV-GFP	CCGATAAAATAAAAGATTTTATTTAGTCTCCAGAAAAAGGGGGGAATGAAAGACCCCACCTGTAGGTTTGGCAAGCTAGCTGCAGTAACGCCATTTTGCAAGGCATGGAAAAATACCAAACC<br>AAGAATAGAGAAGTTCAGATCAAGGGCGGGTACATGAAAATAGCTAACGTTGGGCCAAACAGGATATCTGCGGTGAGCAGTTTCGGCCCCGGCCCGGGGCCAAGAACAGATGGTCACCGC<br>AGTTTCGGCCCCGGCCCGAGGCCAAGAACAGATGGTCCCCAGATATGGCCCAACCCTCAGCAGTTTCTTAAGACCCATCAGATGTTTCCAGGCTCCCCCAAGGACCTGAAATGACCCTGCGC<br>CTTATTTGAATTAACCAATCAGCCTGCTTCTCGCTTCTGTTCGCGCGCTTCTGCTTCCCGAGCTCTATAAAAGAGCTCACAACCCCTCACTCGGCGCGCCAGTCCTCCGACAGACTGAGTCGCC<br>CGGGGGGGTACCGAGCTCTTCGAAGGATCCATCGCCACCATGCCCGCCATGAAGATCGAGTGCCGCATCACCGGCACCCTGAACGGCGTGGAGTTCGAGCTGGTGGGCGGCGGAGAGGG<br>CACCCCCGAGCAGGGCCGCATGACCAACAAGATGAAGAGCACCAAAGGCGCCCTGACCTTCAGCCCCTACCTGCTGAGCCACGTGATGGGCTACGGCTTCTACCACTTCGGCACCTACCCC<br>AGCGGCTACGAGAACCCCTTCCTGCACGCCATCAACAACGGCGGCTACACCAACACCCGCATCGAGAAGTACGAGGACGGCGGCGTGCTGCACGTGAGCTTCAGCTACCGCTACGAGGCC<br>GGCCGCGTGATCGGCGACTTCAAGGTGGTGGGCACCGGCTTCCCCGAGGACAGCGTGATCTTCACCGACAAGATCATCCGCAGCAACGCCACCGTGGAGCACCTGCACCCCATGGGCGAT<br>AACGTGCTGGTGGGCAGCTTCGCCCGCACCTTCAGCCTGCGCGACGGCGGCTACTACAGCTTCGTGGTGGACAGCCACATGCACTTCAAGAGCGCCATCCACCCCAGCATCCTGCAGAACG<br>GGGGCCCCATGTTCGCCTTCCGCCGCGTGGAGGAGCTGCACAGCAACACCGAGCTGGGCATCGTGGAGTACCAGCACGCCTTCAAGACCCCCATCGCCTTCGCCAGATCTCGAGTCTAGCT<br>CGAGGGCGCGCCCGCTGATCAGCCTCGACCTGTGCCTTCTAGTTGCCAGCCATCTGTTGTTTGCCCCTCCCCCGTGCCTTCCTTGACCCTGGAAGGTGCCACTCCCACTGTCCTTTCCTAATA<br>AAATGAGGAAATTGCATCGCATTGTCTGAGTAGGTGTCATTCTATTCTGGGGGGTGGGGTGGGGCAGGACAGCAAGGGGGAGGATTGGGAAGACAATAGCAGGCATGCTGGGGATGCGG<br>TGGGCTCTATGGCTTCTGAGGCGGAAAGAACGTTTCGCGCCATTACTATGTTCGGGCCGTCCTGCATCTCCTGTGAGTCCCCGTCCCGCACCCCCTCTAGGGCTCAGGAGGGCTTGGAGCC<br>GACCCTCCCCACTGTCCCACCGGCCGGGCTGCCTGGACAGGAGCCACCCCCACTTACCTCAGTGTTTTTCCAAACAAAAATTCGGGTCCCTGGCTCTGGCAGGGCCTGTGTCTGCTGTCTAG<br>TGTGCAGGATTTGTAAGGATCCACTCCAAATCCGAGGAGCTCCGATCCGTCGCCAGGGTCTGGGGTCAGCATGCACTGCTGGGGGGTCTTGCCTGGGCTTCCTGGTAGGGTGGAGGGTTC<br>CGTGCTGTGTGTCTGCTGGTTACTGCCGGCTGAGGCCAGGACACCCACACGTGGCTGCTTCCTCCGAGCAAGTGCACC
Correction (IDLV/AAV)	ATGGCTTCAGTGTGGCGGGCGTGTTTGTGTGCCTGCTGGAGTACCCCCGGGGGAAGAGGAAGAAGGGCTCCACCATGGAGCGCTGGTGAGTCTCCTCCTGCTCTGGGGTCTCTCCGGGGG<br>CTGCGGGGCCCAGGCAGGGCTCACAGGGTTGGGTGGAGCTTGGTTTCTCACTTGGAGGCTCCGGAACCAACCCTTTGGTGCTTGTGGGTAAACCAAGGCCGGTGCCTGCCCGGTGTGTTTT<br>GTGGGAGGAAAGAGGCCTGGGTGCCCTGGGGTGGTCAGCAGGGCAGCAAAGGAGTCCCGAGTGGGAGAGGCCCAGCCGCGCCGTCTCGCCTTCCTCCCTCCCCCAGGGGACAGAAGTA<br>CATGACCGCCGTGGTGAAGCTGTTCGGGCCCTTTACCAGGAACTATTACGTGAGAGCTGTGTTACACTTATTGTGAGTCCCCGTCCCGCACCCCCTCTAGGGCTCAGGAGGGCTTGGAGCCG<br>ACCCTCCCCACTGTCCCACCGGCCGGGCTGCCTGGACAGGAGCCACCCCCACTTACCTCAGTGTTTTTCCAAACAAAAATTCGGGTCCCTGGCTCTGGCAGGGCCTGTGTCTGCTGTCTAGT<br>GTGCAGGATTTGTAAGGATCCACTCCAAATCCGAGGAGCTCCGATCCGTCGCCAGGGTCTGGGGTCAGCATGCACTGCTGGGGGGTCTTGCCTGGGCTTCCTGGTAGGGTGGAGGGTTCC<br>GTGCTGTGTGTCTGCTGGTTACTGCCGGCTGAGGCCAGGACACCCACACGTGGCTGCTTCCTCCGAGCAAGTGCACCAGAGCCGCTAGTGTCCACATCAAGGCTGAGAA
sense ssODN	CCAGGCAGCCCGGCCGGTGGGACAGTGGGGAGGGTCGGCTCCAAGCCCTCCTGAGCCCTAGAGGGGGTGCGGGACGGGGACTCACAATAAGTGTAACACAGCTCTCACGTAATAGTTCC<br>TGGTAAAGGGCCCGAACAGCTTCACCACGGCGGTCATGTACTTCTGTCCCCTGGGGGAGGGAGGAAGGCGAGACGGCGCGG
antisense ssODN	CCGCGCCGTCTCGCCTTCCTCCCTCCCCCAGGGGACAGAAGTACATGACCGCCGTGGTGAAGCTGTTCGGGCCCTTTACCAGGAACTATTACGTGAGAGCTGTGTTACACTTATTGTGAGTC<br>CCCGTCCCGCACCCCCTCTAGGGCTCAGGAGGGCTTGGAGCCGACCCTCCCCACTGTCCCACCGGCCGGGCTGCCTGG
sg2_correction	AAGCAAGCATAAAAGGTATAATCTGGGAGATGAATAAACAGGTATTTATTGTAAATTTCCTTCAACTTGTCTTTATGTTTGAACATTTTAATAATAAAATGTTGAATTAGGGCAAAAGGTCAATTT<br>CAGATGAAAACAAAGGGTCCTAGCTAGCCCTAAAGGAAAACCGTTGGCTCTGAAGGACCTTCCTGTACAGATCACTTGGTGTCTCTGGTGTGTGGCCTCATGCTAAGAACCTTGGGGAAGTG<br>GGGACAGGGCATATTCTGTGCTCAAAAAAGTCACTCTGCTCCCTTTCCCGCCTCTTCTAGTCAGCACTGGCACTGGCCAGGGCCCCTGCAGCCTGCCTGAATTTCAACTGCATGCTGATTCTC<br>TTGCCAGTCTGTCGAAATCTGCTGTCCTTCCTCAGGGGTTCCAGTGCGGTAAGAGAAAATGTTTTACTAAGTTCCTCTAATTTTCAAAGGCCATCAAGCAAAATGCCCTTTTTTAGGTAAAAACA<br>AATGAATGATTTGGGAGGATTCCAGCTTCTGTAGAATACTCATGAGCCAAGCCATTATCTAGTATATTTCTGTGCTATTTGGGGATGGAGCTCCCTAAGACAACTATTTGGAAAACAAATTTTG<br>AAAAAACACACAAAACCACCTATACCTCAACAGTAGCAATGAAGTAGCACACACGTACACGTTTACACACACACACATATATATATGATAGATATGCATATATACAGAGCTTTAGGAAGTTGAC<br>ATGTGATATCTGGGAGTTAATCTTTCAGTGTCATTCAAAAAAAATTTAAGTCAGAGGAAATATCTTCTGTGGATAA
sg3_correction	AAGCAAGCATAAAAGGTATAATCTGGGAGATGAATAAACAGGTATTTATTGTAAATTTCCTTCAACTTGTCTTTATGTTTGAACATTTTAATAATAAAATGTTGAATTAGGGCAAAAGGTCAATTT<br>CAGATGAAAACAAAGGGTCCTAGCTAGCCCTAAAGGAAAACCGTTGGCTCTGAAGGACCTTCCTGTACAGATCACTTGGTGTCTCTGGTGTGTGGCCTCATGCTAAGAACCTTGGGGAAGTG<br>GGGACAGGGCATATTCTGTGCTCAAAAAAGTCACTCTGCTCCCTTTCCCGCCTCTTCTAGTCAGCACTGGCACTGGCCAGGGCCCCTGCAGCCTGCCTGAATTTCAACTGCATGCTGATTCTC<br>TTGCCAGTCTGTCGAAATCTGCTGTCCTTTCTCCGGGGTTCTAGTGCGGTAAGAGAAAATGTTTTACTAAGTTCCTCTAATTTTCAAAGGCCATCAAGCAAAATGCCCTTTTTTAGGTAAAAACA<br>AATGAATGATTTGGGAGGATTCCAGCTTCTGTAGAATACTCATGAGCCAAGCCATTATCTAGTATATTTCTGTGCTATTTGGGGATGGAGCTCCCTAAGACAACTATTTGGAAAACAAATTTTG<br>AAAAAACACACAAAACCACCTATACCTCAACAGTAGCAATGAAGTAGCACACACGTACACGTTTACACACACACACATATATATATGATAGATATGCATATATACAGAGCTTTAGGAAGTTGAC<br>ATGTGATATCTGGGAGTTAATCTTTCAGTGTCATTCAAAAAAAATTTAAGTCAGAGGAAATATCTTCTGTGGATAA
cDNA	AAGCAAGCATAAAAGGTATAATCTGGGAGATGAATAAACAGGTATTTATTGTAAATTTCCTTCAACTTGTCTTTATGTTTGAACATTTTAATAATAAAATGTTGAATTAGGGCAAAAGGTCAATTT<br>CAGATGAAAACAAAGGGTCCTAGCTAGCCCTAAAGGAAAACCGTTGGCTCTGAAGGACCTTCCTGTACAGATCACTTGGTGTCTCTGGTGTGTGGCCTCATGCTAAGAACCTTGGGGAAGTG<br>GGGACAGGGCATATTCTGTGCTCAAAAAAGTCACTCTGCTCCCTTTCCCGCCTCTTCTAGTCAGCACTGGCACTGGCCAGGGCCCCTGCAGCCTGCCTGAATTTCAACTGCATGCTGATTCTC<br>TTGCCAGTCTGTCGAAATCTGCTGTCCTTCCTGAGAGGCAGCAGCGCCTGTTGTAGCACCAGGGTGAGAAGGCAGCTCGATAGAAACCTGACATTCCACAAGATGGTCGCCTGGATGATCG<br>CCCTGCATAGCGCCATCCATACAATCGCCCACCTGTTCAACGTCGAGTGGTGCGTCAACGCTAGAGTGAACAATTCAGATCCATATTCTGTTGCTTTATCAGAATTAGGTGACCGTCAAAATGA<br>AAGCTACCTGAACTTCGCCAGGAAGAGAATCAAGAACCCAGAAGGTGGTTTATACTTAGCAGTTACTTTATTAGCTGGTATAACAGGTGTGGTGATTACCCTCTGTCTGATCCTGATCATTACC<br>AGCAGCACAAAGACAATTAGAAGAAGCTATTTCGAGGTGTTCTGGTATACCCACCACCTGTTCGTCATCTTCTTCATCGGACTGGCTATTCATGGTGCAGAACGTATAGTTCGAGGTCAGACT<br>GCTGAGTCTTTAGCAGTTCATAACATCACCGTGTGCGAGCAGAAGATTAGCGAGTGGGGCAAGATCAAAGAGTGTCCCATTCCACAGTTTGCAGGTAACCCACCAATGACATGGAAATGGAT<br>TGTTGGACCAATGTTCCTCTACCTGTGCGAAAGACTGGTCAGATTCTGGAGAAGCCAGCAGAAGGTGGTGATTACGAAGGTGGTCACGCACCCGTTCAAAACGATCGAGCTCCAGATGAAG<br>AAGAAGGGCTTTAAGATGGAAGTGGGCCAGTATATCTTCGTGAAATGTCCCAAAGTCAGCAAACTCGAATGGCATCCCTTCACCCTCACCAGCGCTCCCGAAGAGGACTTCTTTAGCATTCAC<br>ATTAGAATTGTGGGCGATTGGACCGAAGGCCTCTTTAACGCCTGCGGATGCGACAAACAAGAATTCCAAGATGCTTGGAAATTACCAAAGATTGCTGTAGATGGTCCATTTGGTACAGCAAG<br>CGAGGACGTCTTTTCCTATGAGGTTGTTATGTTAGTTGGTGCTGGTATAGGTGTTACTCCATTCGCTTCTATCCTGAAAAGCGTCTGGTACAAATATTGCAATAACGCGACGAATCTGAAGCTC<br>AAGAAAATCTACTTCTACTGGCTCTGTAGAGATACCCACGCTTTCGAATGGTTCGCCGACCTCCTCCAGCTCCTCGAGTCGCAGATGCAGGAACGCAACAATGCGGGGTTCCTCTCGTACAAC<br>ATCTACCTGACCGGATGGGACGAAAGCCAAGCTAACCATTTCGCCGTCCATCACGACGAAGAAAAGGACGTCATTACCGGACTCAAGCAGAAAACCCTGTACGGCAGACCTAATTGGGACA<br>ACGAGTTTAAAACCATAGCTTCTCAACACCCAAATACTAGAATTGGTGTATTCTTATGTGGTCCAGAAGCTCTGGCCGAGACACTCAGCAAGCAGTCCATTAGCAATAGCGAAAGCGGACCCA<br>GAGGCGTCCACTTTATCTTTAATAAAGAGAACTTCTAACATATGTAATCAACCTCTGGATTACAAAATTTGTGAAAGATTGACTGGTATTCTTAACTATGTTGCTCCTTTTACGCTATGTGGATAC<br>GCTGCTTTAATGCCTTTGTATCATGCTATTGCTTCCCGTATGGCTTTCATTTTCTCCTCCTTGTATAAATCCTGGTTGCTGTCTCTTTATGAGGAGTTGTGGCCCGTTGTCAGGCAACGTGGCGT<br>GGTGTGCACTGTGTTTGCTGACGCAACCCCCACTGGTTGGGGCATTGCCACCACCTGTCAGCTCCTTTCCGGGACTTTCGCTTTCCCCCTCCCTATTGCCACGGCGGAACTCATCGCCGCCTG<br>CCTTGCCCGCTGCTGGACAGGGGCTCGGCTGTTGGGCACTGACAATTCCGTGGTGTTGTCGGGGAAGCTGACGTCCTTTCCATGGCTGCTCGCCTGTGTTGCCACCTGGATTCTGCGCGGG<br>ACGTCCTTCTGCTACGTCCCTTCGGCCCTCAATCCAGCGGACCTTCCTTCCCGCGGCCTGCTGCCGGCTCTGCGGCCTCTTCCGCGTCTTCGCCTTCGCCCTCAGACGAGTCGGATCTCCCTT<br>TGGGCCGCCTCCCCGCCATATGTAGATCCCCCGGGGGATCAGCCTCGACTGTGCCTTCTAGTTGCCAGCCATCTGTTGTTTGCCCCTCCCCCGTGCCTTCCTTGACCCTGGAAGGTGCCACTC<br>CCACTGTCCTTTCCTAATAAAATGAGGAAATTGCATCGCATTGTCTGAGTAGGTGTCATTCTATTCTGGGGGGTGGGGTGGGGCAGGACAGCAAGGGGGAGGATTGGGAAGACAATAGCAG<br>GCATGCTGGGGATGCGGTGGGCTCTATGGCAGGGGTTCCAGTGCGGTAAGAGAAAATGTTTTACTAAGTTCCTCTAATTTTCAAAGGCCATCAAGCAAAATGCCCTTTTTTAGGTAAAAACAA<br>ATGAATGATTTGGGAGGATTCCAGCTTCTGTAGAATACTCATGAGCCAAGCCATTATCTAGTATATTTCTGTGCTATTTGGGGATGGAGCTCCCTAAGACAACTATTTGGAAAACAAATTTTGA<br>AAAAACACACAAAACCACCTATACCTCAACAGTAGCAATGAAGTAGCACACACGTACACGTTTACACACACACACATATATATATGATAGATATGCATATATACAGAGCTTTAGGAAGTTGACA<br>TGTGATATCTGGGAGTTAATCTTTCAGTGTCATTCAAAAAAAATTTAAGTCAGAGGAAATATCTTCTGTGGATAA
cDNA-eGFP	AAGCAAGCATAAAAGGTATAATCTGGGAGATGAATAAACAGGTATTTATTGTAAATTTCCTTCAACTTGTCTTTATGTTTGAACATTTTAATAATAAAATGTTGAATTAGGGCAAAAGGTCAATTT<br>CAGATGAAAACAAAGGGTCCTAGCTAGCCCTAAAGGAAAACCGTTGGCTCTGAAGGACCTTCCTGTACAGATCACTTGGTGTCTCTGGTGTGTGGCCTCATGCTAAGAACCTTGGGGAAGTG<br>GGGACAGGGCATATTCTGTGCTCAAAAAAGTCACTCTGCTCCCTTTCCCGCCTCTTCTAGTCAGCACTGGCACTGGCCAGGGCCCCTGCAGCCTGCCTGAATTTCAACTGCATGCTGATTCTC<br>TTGCCAGTCTGTCGAAATCTGCTGTCCTTCCTGAGAGGCAGCAGCGCCTGTTGTAGCACCAGGGTGAGAAGGCAGCTCGATAGAAACCTGACATTCCACAAGATGGTCGCCTGGATGATCG<br>CCCTGCATAGCGCCATCCATACAATCGCCCACCTGTTCAACGTCGAGTGGTGCGTCAACGCTAGAGTGAACAATTCAGATCCATATTCTGTTGCTTTATCAGAATTAGGTGACCGTCAAAATGA<br>AAGCTACCTGAACTTCGCCAGGAAGAGAATCAAGAACCCAGAAGGTGGTTTATACTTAGCAGTTACTTTATTAGCTGGTATAACAGGTGTGGTGATTACCCTCTGTCTGATCCTGATCATTACC<br>AGCAGCACAAAGACAATTAGAAGAAGCTATTTCGAGGTGTTCTGGTATACCCACCACCTGTTCGTCATCTTCTTCATCGGACTGGCTATTCATGGTGCAGAACGTATAGTTCGAGGTCAGACT<br>GCTGAGTCTTTAGCAGTTCATAACATCACCGTGTGCGAGCAGAAGATTAGCGAGTGGGGCAAGATCAAAGAGTGTCCCATTCCACAGTTTGCAGGTAACCCACCAATGACATGGAAATGGAT<br>TGTTGGACCAATGTTCCTCTACCTGTGCGAAAGACTGGTCAGATTCTGGAGAAGCCAGCAGAAGGTGGTGATTACGAAGGTGGTCACGCACCCGTTCAAAACGATCGAGCTCCAGATGAAG<br>AAGAAGGGCTTTAAGATGGAAGTGGGCCAGTATATCTTCGTGAAATGTCCCAAAGTCAGCAAACTCGAATGGCATCCCTTCACCCTCACCAGCGCTCCCGAAGAGGACTTCTTTAGCATTCAC<br>ATTAGAATTGTGGGCGATTGGACCGAAGGCCTCTTTAACGCCTGCGGATGCGACAAACAAGAATTCCAAGATGCTTGGAAATTACCAAAGATTGCTGTAGATGGTCCATTTGGTACAGCAAG<br>CGAGGACGTCTTTTCCTATGAGGTTGTTATGTTAGTTGGTGCTGGTATAGGTGTTACTCCATTCGCTTCTATCCTGAAAAGCGTCTGGTACAAATATTGCAATAACGCGACGAATCTGAAGCTC<br>AAGAAAATCTACTTCTACTGGCTCTGTAGAGATACCCACGCTTTCGAATGGTTCGCCGACCTCCTCCAGCTCCTCGAGTCGCAGATGCAGGAACGCAACAATGCGGGGTTCCTCTCGTACAAC<br>ATCTACCTGACCGGATGGGACGAAAGCCAAGCTAACCATTTCGCCGTCCATCACGACGAAGAAAAGGACGTCATTACCGGACTCAAGCAGAAAACCCTGTACGGCAGACCTAATTGGGACA<br>ACGAGTTTAAAACCATAGCTTCTCAACACCCAAATACTAGAATTGGTGTATTCTTATGTGGTCCAGAAGCTCTGGCCGAGACACTCAGCAAGCAGTCCATTAGCAATAGCGAAAGCGGACCCA<br>GAGGCGTCCACTTTATCTTTAATAAAGAGAACTTCGGATCCGGCGCAACAAACTTCTCTCTGCTGAAACAAGCCGGAGATGTCGAAGAGAATCCTGGACCGGTGAGCAAGGGCGAGGAGCT<br>GTTCACCGGGGTGGTGCCCATCCTGGTCGAGCTGGACGGCGACGTAAACGGCCACAAGTTCAGCGTGTCTGGCGAGGGCGAGGGCGATGCCACCTACGGCAAGCTGACCCTGAAGTTCAT<br>CTGCACCACCGGCAAGCTGCCCGTGCCCTGGCCCACCCTCGTGACCACCCTGACCTACGGCGTGCAGTGCTTCAGCCGCTACCCCGACCACATGAAGCAGCACGACTTCTTCAAGTCCGCC<br>ATGCCCGAAGGCTACGTCCAGGAGCGCACCATCTTCTTCAAGGACGACGGCAACTACAAGACCCGCGCCGAGGTGAAGTTCGAGGGCGACACCCTGGTGAACCGCATCGAGCTGAAGGGC<br>ATCGACTTCAAGGAGGACGGCAACATCCTGGGGCACAAGCTGGAGTACAACTACAACAGCCACAACGTCTATATCATGGCCGACAAGCAGAAGAACGGCATCAAGGCGAACTTCAAGATCC<br>GCCACAACATCGAGGACGGCAGCGTGCAGCTCGCCGACCACTACCAGCAGAACACCCCCATCGGCGACGGCCCCGTGCTGCTGCCCGACAACCACTACCTGAGCACCCAGTCCGCCCTGA<br>GCAAAGACCCCAACGAGAAGCGCGATCACATGGTCCTGCTGGAGTTCGTGACCGCCGCCGGGATCACTCTCGGCATGGACGAGCTGTACAAGTAGCATATGTAATCAACCTCTGGATTACA<br>AAATTTGTGAAAGATTGACTGGTATTCTTAACTATGTTGCTCCTTTTACGCTATGTGGATACGCTGCTTTAATGCCTTTGTATCATGCTATTGCTTCCCGTATGGCTTTCATTTTCTCCTCCTTGTA<br>TAAATCCTGGTTGCTGTCTCTTTATGAGGAGTTGTGGCCCGTTGTCAGGCAACGTGGCGTGGTGTGCACTGTGTTTGCTGACGCAACCCCCACTGGTTGGGGCATTGCCACCACCTGTCAGC<br>TCCTTTCCGGGACTTTCGCTTTCCCCCTCCCTATTGCCACGGCGGAACTCATCGCCGCCTGCCTTGCCCGCTGCTGGACAGGGGCTCGGCTGTTGGGCACTGACAATTCCGTGGTGTTGTCG<br>GGGAAGCTGACGTCCTTTCCATGGCTGCTCGCCTGTGTTGCCACCTGGATTCTGCGCGGGACGTCCTTCTGCTACGTCCCTTCGGCCCTCAATCCAGCGGACCTTCCTTCCCGCGGCCTGCT<br>GCCGGCTCTGCGGCCTCTTCCGCGTCTTCGCCTTCGCCCTCAGACGAGTCGGATCTCCCTTTGGGCCGCCTCCCCGCCATATGTAGATCCCCCGGGGGATCAGCCTCGACTGTGCCTTCTAG<br>TTGCCAGCCATCTGTTGTTTGCCCCTCCCCCGTGCCTTCCTTGACCCTGGAAGGTGCCACTCCCACTGTCCTTTCCTAATAAAATGAGGAAATTGCATCGCATTGTCTGAGTAGGTGTCATTCTA<br>TTCTGGGGGGTGGGGTGGGGCAGGACAGCAAGGGGGAGGATTGGGAAGACAATAGCAGGCATGCTGGGGATGCGGTGGGCTCTATGGCAGGGGTTCCAGTGCGGTAAGAGAAAATGTT<br>TTACTAAGTTCCTCTAATTTTCAAAGGCCATCAAGCAAAATGCCCTTTTTTAGGTAAAAACAAATGAATGATTTGGGAGGATTCCAGCTTCTGTAGAATACTCATGAGCCAAGCCATTATCTAGT<br>ATATTTCTGTGCTATTTGGGGATGGAGCTCCCTAAGACAACTATTTGGAAAACAAATTTTGAAAAAACACACAAAACCACCTATACCTCAACAGTAGCAATGAAGTAGCACACACGTACACGTT<br>TACACACACACACATATATATATGATAGATATGCATATATACAGAGCTTTAGGAAGTTGACATGTGATATCTGGGAGTTAATCTTTCAGTGTCATTCAAAAAAAATTTAAGTCAGAGGAAATATCT
CYBB_c252G>A_ssODN	CTCTTGCCAGTCTGTCGAAATCTGCTGTCCTTCCTCAGGGGTTCCAGTGCAGTAAGAGAAAATGTTTTACTAAGTTCCTCTAATTTTCAAAGGCCATCAA

Supplementary Table 3. Flow cytometry staining panels

Panel	Antibody/Reagent	Flurochrome	Supplier	Cat. No	Clone	Volume per 100 uL or 1 mio cells
Immunophenotype	PE anti-human CD34	PE	BD	555822	581	6 µL
	FITC anti-human CD45	FITC	BD	345808	2D1	3 µL
	BV605 anti-human CD38	BV605	BD	562665	HB7	2 µL
	PE-Cy7 anti-human CD90	PE-Cy7	BD	561558	5E10	2 µL
	eFlour450 anti-human CD45RA	eFlour450	BD	747645	HI100	2 µL
	LIVE/DEAD™ Fixable Near-IR (1:10 dilution)		Thermo Fisher	L34976		1 uL
Mice engraftment analysis	Human TruStain FcX		Biolegend	422301		5 uL
	APC-Cy7 anti-human HLA_A,B,C	APC-Cy7	BioLegend	311426	W6/32	3 uL
	PE anti-human CD33	PE	eBioscience	12-0338-42	WM53	3 uL
	eFlour450 anti-human CD45	eFlour450	eBioscience	15259564/48-0459-42	HI30	3 uL
	APC anti-human CD19	APC	BD	555415	HIB19	6 uL
	PE-Cy5 anti-mouse TER-119	PE-Cy5	eBioscience	15-5921-82	TER-119	0.8 uL
	PE-Cy7 anti-mouse CD45.1	PE-Cy7	TonboBio	60-0453-U100	A20	1.5 uL
	Propidium Iodide (PI) (1:20 dilution)					1 uL
	Human TruStain FcX		Biolegend	422301		5 uL
Granulocyte differentiation	PE anti-human CD34	PE	BD	555822	581	6 µL
	FITC anti-human CD15	FITC	BioLegend	301903	HI98	2 uL
DHR assay	PE anti-human CD15	PE	BioLegend	301905	HI98	3 uL
	Dihydrorhodamine-123		Sigma			15 ng
Viability/ proliferation	Propidium Iodide (PI) (1:20 dilution)					1 uL
FACS mice bone marrow	eFlour450 anti-human CD45	eFlour450	eBioscience	15259564/48-0459-42	HI30	1 uL
	FITC anti-human CD34	FITC	BioLegend	343503	581	1 uL
	APC-Cy7 anti-human CD10	APC-Cy7	BioLegend	312212	HI10a	0.2 uL
	Propidium Iodide (PI) (1:20 dilution)					1 uL

Supplementary Table 4. Primer sequences

Name	Sequence 5' --> 3'
CYBA_sanger_F	TTGGTTTCTCACTTGGAGGC
CYBA_sanger_R	CACCAGTAGGTAGATGCCG
CYBA_TruSeq_F	ACACTCTTTCCCTACACGACGCTCTTCCGATCTCAGCCGCGCCGTCTCG
CYBA_TruSeq_R	GTGACTGGAGTTCAGACGTGTGCTCTTCCGATCTGGTGGCTCCTGTCCAGGCA
CYBB_c.252_sanger_F	AAACCGTTGGCTCTGAAGGA
CYBB_c.252_sanger_R	TGCTCCCCAGTTCCATGAAG
CYBB_c.252_TruSeq_F	ACACTCTTTCCCTACACGACGCTCTTCCGATCTTCTTCTAGTCAGCACTGGCACT
CYBB_c.252_TruSeq_R	GTGACTGGAGTTCAGACGTGTGCTCTTCCGATCTTTCTACAGAAGCTGGAATCCTCCC
CYBB_cDNA_ddPCR_F	ACAGGAATGGATAGATAGAATGTGA
CYBB_cDNA_ddPCR_R	CTCACCCTGGTGCTACAACA
CYBB_cDNA_ddPCR_probe	FAM/ATGCCCTGTCCCCACTTCCCCAAGGT/ IB™FQ
Alb_ddPCR_F	GCTGTCATCTCTTGTGGGCTGT
Alb_ddPCR_R	ACTCATGGGAGCTGCTGGTTC
Alb_ddPCR_probe	HEX/CCTGTCATGCCCACACAAATCTCTCC/BHQ
**CAST-seq**	
**Name**	**Sequence 5' --> 3'**
bait	ATCCTCCCAAATCATTCATTTG
nested	GACTGGAGTTCAGACGTGTGCTCTTCCGATCTAAGGGCATTTTGCTTGATGGC
decoy1	GCCAGTCTGTCGAAATCTG
decoy2	GAGAATCAGCATGCAGTTG
decoy3	CCAGTGCTGACTAGAAGAG

Supplementary Table 5. Setup information of NOG mice engraftments (sg2 vs sg3)

Condition	Mouse #	Cells treated	Cells after expansion	Viability after expansion	Fold expansion	Resuspension volume	Injected volume	Cells injected
Mock	1	2.00E+06	5.39E+06	96.53%	2.695	210 µL	190 µL	4.88E+06
	2	2.00E+06	5.15E+06	95.96%	2.575	210 µL	190 µL	4.66E+06
	3	2.00E+06	5.17E+06	95.62%	2.585	210 µL	190 µL	4.68E+06
	4	2.00E+06	5.08E+06	96.21%	2.54	210 µL	190 µL	4.60E+06
sg2	5	2.00E+06	5.64E+06	96.23%	2.82	210 µL	190 µL	5.10E+06
	6	2.00E+06	5.49E+06	93.23%	2.745	210 µL	190 µL	4.97E+06
	7	2.00E+06	5.63E+06	95.93%	2.815	210 µL	190 µL	5.09E+06
	8	2.00E+06	5.54E+06	94.87%	2.77	210 µL	190 µL	5.01E+06
sg3	9	2.00E+06	2.97E+06	88.06%	1.485	210 µL	190 µL	2.69E+06
	10	2.00E+06	2.86E+06	86.14%	1.43	210 µL	190 µL	2.59E+06
	11	2.00E+06	2.93E+06	88.03%	1.465	210 µL	190 µL	2.65E+06
	12	2.00E+06	3.13E+06	90.49%	1.565	210 µL	190 µL	2.83E+06

Supplementary Table 6. Setup information of NOG mice engraftments (STD Cas9 vs HiFi Cas9)

Condition	Mouse #	Cells treated	Cells after expansion	Viability after expansion	Fold expansion	Resuspension volume	Injected volume	Cells injected
STDCas9 correction	1	2.00E+06	3.08E+06	88.86%	1.54	210 µL	190 µL	2.79E+06
Mock	2	2.00E+06	4.54E+06	96.47%	2.27	210 µL	190 µL	4.11E+06
Mock	3	2.00E+06	4.50E+06	95.65%	2.25	210 µL	190 µL	4.07E+06
Mock	4	2.00E+06	5.11E+06	97.32%	2.56	210 µL	190 µL	4.62E+06
HiFi Cas9correction	5	2.00E+06	5.03E+06	94.47%	2.52	210 µL	190 µL	4.55E+06
STDCas9 correction	6	2.00E+06	3.13E+06	89.96%	1.57	210 µL	190 µL	2.83E+06
HiFi Cas9correction	7	2.00E+06	5.06E+06	94.08%	2.53	210 µL	190 µL	4.58E+06
STDCas9 correction	8	2.00E+06	2.88E+06	91.44%	1.44	210 µL	190 µL	2.61E+06
HiFi Cas9 cDNA-eGFP	9	2.00E+06	3.56E+06	91.47%	1.78	210 µL	190 µL	3.22E+06
HiFi Cas9 cDNA-eGFP	10	2.00E+06	3.65E+06	92.55%	1.83	210 µL	190 µL	3.30E+06
HiFi Cas9correction	11	2.00E+06	5.14E+06	95.19%	2.57	210 µL	190 µL	4.65E+06
HiFi Cas9 cDNA-eGFP	12	2.00E+06	3.55E+06	94.78%	1.78	210 µL	190 µL	3.21E+06

Supplementary Table 7. HSPC donors

HSPC donor	Age (years)	Sex
Healthy donor 1	24	Male
Healthy donor 2	23	Female
Healthy donor 3	29	Male
Healthy donor 4	24	Male
CYBB-HSPCs donor 1	34	Female
	Mean = 26.8	

Supplementary References

1. Roos, D. *et al.* Hematologically important mutations: X-linked chronic granulomatous disease (fourth update). *Blood Cells Mol Dis* **90**, 102587 (2021). https://doi.org:10.1016/j.bcmd.2021.102587
