# Supplementary material for: Targeted gene editing and near-universal cDNA insertion of CYBA and CYBB as a treatment for chronic granulomatous disease
Source: Nat Commun. 2025 Aug 12;16:7475. doi: 10.1038/s41467-025-62738-2 (PMC12343970; doi:10.1038/s41467-025-62738-2)
Supplement: Supplementary file 2 — Description of Additional Supplementary Files [file 41467_2025_62738_MOESM2_ESM.pdf]

Description of Additional Supplementary Files

File Name: Supplementary Data 1

Description: Raw output of DISCOVER-seq analysis.

File Name: Supplementary Data 2

Description: Raw output of the CAST-seq analysis.
