# Supplementary material for: Targeted gene editing and near-universal cDNA insertion of CYBA and CYBB as a treatment for chronic granulomatous disease
Source: Nat Commun. 2025 Aug 12;16:7475. doi: 10.1038/s41467-025-62738-2 (PMC12343970; doi:10.1038/s41467-025-62738-2)
Supplement: Supplementary file 5 — Reporting Summary [file 41467_2025_62738_MOESM5_ESM.pdf]

nature portfolio

Corresponding author(s): Prof. Jacob Giehm Mikkelsen, PhD

Last updated by author(s): Jul 24, 2025

Reporting Summary

Nature Portfolio wishes to improve the reproducibility of the work that we publish. This form provides structure for consistency and transparency in reporting. For further information on Nature Portfolio policies, see our Editorial Policies and the Editorial Policy Checklist.

Statistics

For all statistical analyses, confirm that the following items are present in the figure legend, table legend, main text, or Methods section.

n/a	Confirmed	
☐	☒	The exact sample size (*n*) for each experimental group/condition, given as a discrete number and unit of measurement
☐	☒	A statement on whether measurements were taken from distinct samples or whether the same sample was measured repeatedly
☐	☒	The statistical test(s) used AND whether they are one- or two-sided<br>*Only common tests should be described solely by name; describe more complex techniques in the Methods section.*
☒	☐	A description of all covariates tested
☐	☒	A description of any assumptions or corrections, such as tests of normality and adjustment for multiple comparisons
☐	☒	A full description of the statistical parameters including central tendency (e.g. means) or other basic estimates (e.g. regression coefficient) AND variation (e.g. standard deviation) or associated estimates of uncertainty (e.g. confidence intervals)
☐	☒	For null hypothesis testing, the test statistic (e.g. *F*, *t*, *r*) with confidence intervals, effect sizes, degrees of freedom and *P* value noted<br>*Give P values as exact values whenever suitable.*
☒	☐	For Bayesian analysis, information on the choice of priors and Markov chain Monte Carlo settings
☒	☐	For hierarchical and complex designs, identification of the appropriate level for tests and full reporting of outcomes
☒	☐	Estimates of effect sizes (e.g. Cohen's *d*, Pearson's *r*), indicating how they were calculated

Our web collection on statistics for biologists contains articles on many of the points above.

Software and code

Policy information about availability of computer code

Data collection	NovoExpress v. 1.5.6., Illumina iSeq 100, Illumina NovaSeq 6000, QX Manager Software Standard Edition 2.2
Data analysis	FlowJo v. 10.8.2. NovoExpress v. 1.5.6. CRISPResso2. Prism 10 GraphPad software. QX Manager Software Standard Edition 2.2, BLENDER2, CAST-Seq Bioinformatic pipeline

For manuscripts utilizing custom algorithms or software that are central to the research but not yet described in published literature, software must be made available to editors and reviewers. We strongly encourage code deposition in a community repository (e.g. GitHub). See the Nature Portfolio guidelines for submitting code & software for further information.

Data

Policy information about availability of data

All manuscripts must include a data availability statement. This statement should provide the following information, where applicable:

- Accession codes, unique identifiers, or web links for publicly available datasets
- A description of any restrictions on data availability
- For clinical datasets or third party data, please ensure that the statement adheres to our policy

All data supporting the findings of this study are available within the paper and its supplementary information files. Raw sequencing data of CD34+ HSPCs are deposited at the European Genome-Phenome Archive (EGA), which is hosted by the European Bioinformatics Institute and the Center for Genomic Regulation. The data are available under the accession number EGAS50000001155 under controlled access at EGA due to privacy laws and legal restrictions associated with sharing

sensitive data under the General Data Protection Regulation (GDPR). DISCOVER-seq data generated in this study have been deposited to NCBI's Gene Expression Omnibus and are accessible through GEO Series accession number GSE287370 [https://www.ncbi.nlm.nih.gov/geo/query/acc.cgi?acc=GSE287370]. Source data are provided with this paper.

Research involving human participants, their data, or biological material

Policy information about studies with human participants or human data. See also policy information about sex, gender (identity/presentation), and sexual orientation and race, ethnicity and racism.

Reporting on sex and gender	n/a
Reporting on race, ethnicity, or other socially relevant groupings	n/a
Population characteristics	Voluntary HSPC donors are 23-34 years of age (mean = 26.8).
Recruitment	Mobilized peripheral blood (mPB) CD34+ HSPCs were collected by standard procedures after informed consent from voluntary healthy donors (N = 3 male and N = 1 female) and a heterozygous carrier (N = 1 female) at the Department of Clinical Immunology, Aarhus University Hospital.
Ethics oversight	Mobilized peripheral blood (mPB) CD34+ HSPCs were collected by standard procedures after informed consent from healthy donors (N = 4) and a heterozygous carrier (N = 1) at the Department of Clinical Immunology, Aarhus University Hospital under a study protocol approved by The Central Denmark Region Committees on Health Research Ethics with approval number 1-10-72-144-19

Note that full information on the approval of the study protocol must also be provided in the manuscript.

Field-specific reporting

Please select the one below that is the best fit for your research. If you are not sure, read the appropriate sections before making your selection.

☒ Life sciences ☐ Behavioural & social sciences ☐ Ecological, evolutionary & environmental sciences

For a reference copy of the document with all sections, see nature.com/documents/nr-reporting-summary-flat.pdf

Life sciences study design

All studies must disclose on these points even when the disclosure is negative.

Sample size	Sample size was chosen based on cell and animal availability as well as previous studies and general accepted guidelines in the field. Sample size was not calculated prior to experiments.
Data exclusions	Not data was excluded from the analysis.
Replication	All experiments were conducted using a minimum of 3 biological replicates. All attempts at replication were successful.
Randomization	NOG mice was randomly chosen to receive cells from a given treatment.
Blinding	Blinding was not performed, as this it not the standard within the field.

Reporting for specific materials, systems and methods

We require information from authors about some types of materials, experimental systems and methods used in many studies. Here, indicate whether each material, system or method listed is relevant to your study. If you are not sure if a list item applies to your research, read the appropriate section before selecting a response.

Materials & experimental systems

n/a	Involved in the study
☐	☒ Antibodies
☐	☒ Eukaryotic cell lines
☒	☐ Palaeontology and archaeology
☐	☒ Animals and other organisms
☒	☐ Clinical data
☒	☐ Dual use research of concern
☒	☐ Plants

Methods

n/a	Involved in the study
☒	☐ ChIP-seq
☐	☒ Flow cytometry
☒	☐ MRI-based neuroimaging

Antibodies

Antibodies used

A full list of antibodies, clone, supplier and volumes are found in supplementary table 3

Validation

All antibodies used were validated by the manufacturers/suppliers for use with flow cytometry. Antibodies were additionally titrated prior to use.

Eukaryotic cell lines

Policy information about cell lines and Sex and Gender in Research

Cell line source(s)

K562 (#CCL-243) and HEK293T (#CRL-3216) cells were derived from ATCC. VPC 2.0 cells were derived from Thermo Fisher (#A49784). Mobilized peripheral blood (mPB) CD34+ HSPCs were collected by standard procedures after informed consent from healthy donors (N = 3 male and N=1 female) and a heterozygous carrier (N = 1 female) at the Department of Clinical Immunology, Aarhus University Hospital under a study protocol approved by The Central Denmark Region Committees on Health Research Ethics with approval number 1-10-72-144-19.

Authentication

HSPCs were validated by flow cytometry staining for CD34. Remaining cell lines were authenticated by suppliers and by visual inspection by us upon recieving.

Mycoplasma contamination

Immortalized cell lines were routinely assayed for mycoplasma contamination. Cells with positive mycoplasma contamination was not used for experiments.

Commonly misidentified lines
(See ICLAC register)

No commonly misidentified cell lines were used in the study.

Animals and other research organisms

Policy information about studies involving animals; ARRIVE guidelines recommended for reporting animal research, and Sex and Gender in Research

Laboratory animals

6-8 weeks old female NOG mice (NOD.Cg-Prkdcscid Il2rgtm1Sug/JicTac) were purchaed from Taconic Biosciences

Wild animals

Provide details on animals observed in or captured in the field; report species and age where possible. Describe how animals were caught and transported and what happened to captive animals after the study (if killed, explain why and describe method; if released, say where and when) OR state that the study did not involve wild animals.

Reporting on sex

Female NOG mice only was used for experiments due to common practice.

Field-collected samples

For laboratory work with field-collected samples, describe all relevant parameters such as housing, maintenance, temperature, photoperiod and end-of-experiment protocol OR state that the study did not involve samples collected from the field.

Ethics oversight

All animal experiments were performed under the approval of The Danish Animal Inspectorate (License no. 2018-15-0201-01506 and 2023-15-0201-01458).

Note that full information on the approval of the study protocol must also be provided in the manuscript.

Plants

Seed stocks	*Report on the source of all seed stocks or other plant material used. If applicable, state the seed stock centre and catalogue number. If plant specimens were collected from the field, describe the collection location, date and sampling procedures.*
Novel plant genotypes	*Describe the methods by which all novel plant genotypes were produced. This includes those generated by transgenic approaches, gene editing, chemical/radiation-based mutagenesis and hybridization. For transgenic lines, describe the transformation method, the number of independent lines analyzed and the generation upon which experiments were performed. For gene-edited lines, describe the editor used, the endogenous sequence targeted for editing, the targeting guide RNA sequence (if applicable) and how the editor was applied.*
Authentication	*Describe any authentication procedures for each seed stock used or novel genotype generated. Describe any experiments used to assess the effect of a mutation and, where applicable, how potential secondary effects (e.g. second site T-DNA insertions, mosiacism, off-target gene editing) were examined.*

Flow Cytometry

Plots

Confirm that:

☒ The axis labels state the marker and fluorochrome used (e.g. CD4-FITC).

☒ The axis scales are clearly visible. Include numbers along axes only for bottom left plot of group (a 'group' is an analysis of identical markers).

☒ All plots are contour plots with outliers or pseudocolor plots.

☒ A numerical value for number of cells or percentage (with statistics) is provided.

Methodology

Sample preparation	CD34+ HSPCs for flow cytometry were prepared from ex vivo cultures. For determination of human engraftment in immunodeficient mice, blood, spleen and bone marrow were collected from transplanted mice 16-weeks after injection. Leukocutes were collected using dextran seperation followed by red blood cell lysis (RBCL). Spleens were dissociated by passing through a 70 um filter followed by RBCL. Bone marrow were extracted from the femur and tibia of both back legs of the mice using a 23 gauge needle followed by RBCL. All cells were washed 2x in FACS buffer before staining.
Instrument	NovoCyte 2100YB Analyzer (Agilent) equipped with two lasers (488 nm and 561 nm) or a NovoCyte Quanteon 4025 flow cytometer (Agilent) equipped with four lasers (405 nm, 488 nm, 561 nm, and 637 nm). Flow cytometry assisted cell sorting (FACS) was performed on a Bigfoot cell sorter (Thermo Fisher) equipped with six lasers (349 nm, 405 nm, 488 nm, 561 nm, and 640 nm).
Software	The acquired data were analyzed using NovoExpress v. 1.5.6 or FlowJo v. 10.8.2. Sorting data were analysed using SQS v.1.9.4.
Cell population abundance	Detailed sort reports will be made available upon request from the corresponding author.
Gating strategy	Detailed gating strategies are provided in the supplementary material.

☒ Tick this box to confirm that a figure exemplifying the gating strategy is provided in the Supplementary Information.
